# A guide to machine learning for bacterial host attribution using genome sequence data

**DOI:** 10.1099/mgen.0.000317

**Published:** 2019-11-28

**Authors:** Nadejda Lupolova, Samantha J. Lycett, David L. Gally

**Affiliations:** ^1^​ Division of Infection and Immunity, The Roslin Institute, University of Edinburgh, Easter Bush Campus, Edinburgh, EH25 9RG, UK

**Keywords:** machine learning, host attribution, *Salmonella*, host specificity, whole-genome sequences

## Abstract

With the ever-expanding number of available sequences from bacterial genomes, and the expectation that this data type will be the primary one generated from both diagnostic and research laboratories for the foreseeable future, then there is both an opportunity and a need to evaluate how effectively computational approaches can be used within bacterial genomics to predict and understand complex phenotypes, such as pathogenic potential and host source. This article applied various quantitative methods such as diversity indexes, pangenome-wide association studies (GWAS) and dimensionality reduction techniques to better understand the data and then compared how well unsupervised and supervised machine learning (ML) methods could predict the source host of the isolates. The study uses the example of the pangenomes of 1203 *Salmonella enterica* serovar Typhimurium isolates in order to predict 'host of isolation' using these different methods. The article is aimed as a review of recent applications of ML in infection biology, but also, by working through this specific dataset, it allows discussion of the advantages and drawbacks of the different techniques. As with all such sub-population studies, the biological relevance will be dependent on the quality and diversity of the input data. Given this major caveat, we show that supervised ML has the potential to add real value to interpretation of bacterial genomic data, as it can provide probabilistic outcomes for important phenotypes, something that is very difficult to achieve with the other methods.

## Data Summary

All data was obtained from and is publicly available at EnteroBase: http://enterobase.warwick.ac.uk/. The dataset was previously analysed by our group in 2017.

**Impact Statement**

Inference of phenotype from genotype is an important aim for many biological systems. In the case of bacteria, we now have access to hundreds of thousands of genomes for which evolutionary relationships are being determined and, when allied to valuable metadata about the sequenced isolates, can be used for more predictive purposes. Of particular value for public health is prediction of virulence and source attribution based on genome sequence; for example, how severe might the infection be and from which animal host, and therefore potentially food source or environment, might an isolate originate. This article is a review of specific methods that can be applied to genomic data to make such phenotype predictions, with an emphasis on machine learning, which is now being more widely adopted in conjunction with phylogenetic tools. The review uses a previously published dataset to investigate the potential host animal sources of *Salmonella* *enterica* serovar Typhimurium isolates and is primarily intended as a guide to the different approaches with bacterial genome sequences as the starting point. The article includes discussion of the relative advantages and disadvantages of the methods, as well and more general caveats around dataset quality and over-fitting.

## Introduction

Machine learning (ML) has increased in popularity in data-rich subjects, including the extensive data surrounding genome sequencing and other 'omics' in biological systems [1]. More widely available computing facilities, as well as ML algorithms that can be readily implemented in most programming languages, make ML approaches more accessible for researchers and data enthusiasts. However, it can be difficult for an inexperienced user to choose between the many algorithms available and decide how appropriate these can be to help address different specific questions. This article looks at different methods to analyse and predict bacterial host attribution using *Salmonella* *enterica* serovar Typhimurium genome sequences as the dataset. The work is intended as both a review and research paper, as it introduces and compares different statistical approaches with supervised and unsupervised ML methods.

In this study, whole-genome sequences (WGSs) were obtained from the public database EnteroBase [2]. Such databases [2, 3] are updated daily, with new genomes added to the hundreds of thousands of bacterial genomes already stored. Nevertheless, in order to take full advantage of this resource for analysis of genotype to phenotype relationships, clear and reliable metadata is required, for example location, source and date of isolation should be stored alongside the genomic sequences. It is unfortunate and a block to progress that this is not the case for the majority of the deposited sequence samples. As diagnostic sequencing and metagenomic samples add to these databases, the opportunities to further our fundamental knowledge of these bacteria increase along with our capacity to make predictions of complex phenotypes, such as pathogenic potential and host source. It is appreciated that sometimes these phenotypes are difficult to assign to just one part of the interaction; for example, the likely severity of an infection will be related to many factors not directly associated with the bacterial genome, in particular the genetics and status of the infected host. However, it does not mean that such predictions are not valuable and they could be used to inform treatment or intervention decisions. In a similar way, the capacity to predict the host or environmental source of an isolate from sequence data could be valuable to identify the origins of outbreak strains or sources of contamination, but it can be difficult as bacteria may be isolated from a host in which they are only transient and so it is accepted that the 'labelling' of the host for an isolate may not be correct; as such there is embedded error in the metadata that has to be taken into consideration for any analysis. Possibly, and as a consequence of this complexity, ML offers insights from this data potentially not available from other approaches.

In biology, some ML models have been implemented, for example for predicting macromolecule structure [4], tumour classification [5], reconstruction of gene networks [6]⁠ and virtual drug discovery [7]. In bacterial genomics, examples where ML has been used include prediction of antibiotic resistance [8], solubility of recombinant proteins [9], clarification of taxonomic issues [10], pathogenicity predictions [11], host adaptation [12]⁠ and evaluation of zoonotic potential [13].

The exemplar for this study is *S. enterica* serovar Typhimurium, a bacterium that can infect many different hosts, including birds, pigs, cattle and humans [14]. While we know that certain other *Salmonella* serovars, such as *S *. *enterica* serovar Typhi can be relatively host restricted, *S. enterica* serovar Typhimurium has been considered a 'generalist', able to thrive in multiple hosts from which humans can be infected and become ill [15, 16]. Nevertheless, recent studies have identified more human specific-clades of *S. enterica* serovar Typhimurium [17, 18]⁠, and our own work using a single supervised ML method (support vector machine, SVM) supported the idea that there likely exists both generalists and restricted specialists in the *S. enterica* serovar Typhimurium serovar, and these can be predicted based on gene content [19].

This current study compared the capacity of different popular ML and other statistical approaches to analyse the same set of *S. enterica* serovar Typhimurium genome sequence data as was used in our previous work [19], in order to determine whether genetic signals relating to host 'specificity' or 'restriction' can be identified and how well the different approaches can predict the host from which the isolate was sampled. Complications of the analysis are those often shared with many biological systems and the experiments used to interrogate them: (1) the number of samples is a vanishingly minute representation of the total population structure and so will be, by default, biased to some extent; (2) while the isolation host is known (and is hopefully entered correctly), it does not mean that every isolate potentially has that host as their main preferred habitat.

The working hypothesis is that there will be genetic signals that associate with the different hosts, the expectation is that such signals will vary considerably across the population and may be confounded by strong phylogenetic signals. In some areas, phylogenetic structure due to horizontal gene transfer [20] and mutations as adaptation mechanisms [19] ⁠does align quite well with host restriction [19, 21, 22], so in fact a measure of the success of the different techniques will be how they can identify isolates from different hosts that are within the same phylogenic sub-clusters.

## Introduction to the analysis methods

To distinguish between any populations, multiple characteristics can be used. In ML analyses, these characteristics are called features. Any relevant information can be used, such as the geography of the isolates, time, farming type of the animals from which isolates are collected (industrial, extensive, backyard), antibiotics used or not, etc. In this review, we focus only on genomic features of the isolates. The features extracted from the WGSs can be of different types, for example SNPs in core genes, short sequence regions of a particular length, i.e. *k* bases long (k-mers), whole genes or predicted proteins being present/absent or described by a type. By definition, features are distinctive characteristics of a population and so non-discriminatory information is generally removed as a first step to speed up analyses. The focus is to then use these features to assign relationships between populations and build classifiers for prediction (Table 1). It should be noted that once selected, features can be reduced or engineered. In general, it may be that some of the features have exactly or very nearly the same pattern between the isolates of the population (e.g. all the isolates of that population have either gene 1 allele A+gene 2 allele B, or gene 1 allele C+gene 2 allele D), in which case combining these features might improve the prediction or generalization of the model, or at least speed up the analysis; so this would be an example of feature reduction. ‘Combining’ features can be done simply for features that are correlated (just select one of them), or more sophisticated dimensionality reduction techniques (DRT) can be employed to create a smaller number of particularly informative combined features. By contrast, feature engineering can be used when the model is simple, so that features can be augmented to increase their complexity. For example, a single feature such as a protein variant (PV) represents a single gene, and this sequence could be expanded into multiple features such as k-mers or SNPs, increasing the amount of information for analysis in the model. In this review, we used only the pangenome matrix of predicted proteins as described previously [19] as the features.

**Table 1.** Basic description of data workflow

Whole-genome sequences quality control, mapping to reference, *de-novo* assembly, annotation		
Description	Purpose	Methods
What describes the data?	Features extraction	SNPs, k-mers, proteins, …
What are the most important descriptors?	Features selection	Pan and core GWAS, chi-square, recursive feature elimination algorithms, …
Can descriptors be combined/transformed?	Feature transformation	Scaling and centring, PCA, MDS, t-SNE, auto-encoders, …
Group data by underlying similarities	Unsupervised ML	Phylogeny, k-means, hierarchical clustering, …
Find the hidden patterns in a defined class; classify unknown data	Supervised ML	Random forest, neural networks, SVM, k-nearest-neighbour, …

The underlying structure or distribution of the data can be revealed using unsupervised ML (Table 2). Unsupervised ML groups similar objects together into clusters (i.e. in our case, isolates with similar patterns of predicted proteins – PVs). Examples of unsupervised ML algorithms are k-means clustering, types of hierarchical clustering and latent Dirichlet allocation (LDA) (Table 2). k-means clustering optimizes the assignment into clusters by re-grouping objects until the ‘distances’ (dis-similarity) within clusters are minimized and maximized between clusters. Hierarchical clustering can be divisive and agglomerative, the former starts with a whole dataset progressively dividing it at the most distinct splits, the latter starts by combining the most similar objects first, and then progressively forms bigger groups. We include LDA here because it is a technique used in population genetics to probabilistically assign individuals to populations [23], and is now also commonly used in natural language processing to find ‘topics’ in text documents [24]. Here, it is assumed that each object contains words (genes/PVs) belonging to several topics (populations), and the object is described by its topic profile, but it is not known at the beginning what those topics are.

**Table 2.** Summary of analysis methods with links to tutorials

**Method**		**Tutorial**
**Dimensionality reduction methods**		
1	Principal component analysis (PCA) [43]	PCA tutorial http://www.sthda.com/english/articles/31-principal-component-methods-in-r-practical-guide/112-pca-principal-component-analysis-essentials/
**Unsupervised** **machine learning**		
1	k-means [44]⁠	k-means tutorial https://www.datanovia.com/en/lessons/k-means-clustering-in-r-algorith-and-practical-examples/
2	Agglomerative hierarchical clustering (AHC) [45]	AHC tutorial https://www.datanovia.com/en/lessons/agglomerative-hierarchical-clustering/
3	Divisive hierarchical clustering (DHC) [45]⁠	DHC tutorial https://www.datanovia.com/en/lessons/divisive-hierarchical-clustering/
4	Latent Dirichlet allocation (LDA) [24]⁠	LDA tutorial https://eight2late.wordpress.com/2015/09/29/a-gentle-introduction-to-topic-modeling-using-r/
**Supervised** **machine learning**		
1	Support vector machines (SVMs) [46]⁠	SVM tutorial http://www.sthda.com/english/articles/36-classification-methods-essentials/144-svm-model-support-vector-machine-essentials/
2	Random forest (RF) [47]⁠	RF tutorial http://www.sthda.com/english/articles/35-statistical-machine-learning-essentials/140-bagging-and-random-forest-essentials/
3	Neural network (NN) [48]⁠	NN tutorial http://htmlpreview.github.io/?https://github.com/ledell/sldm4-h2o/blob/master/sldm4-deeplearning-h2o.html

Overall, unsupervised ML is quick, beginner friendly (as there are fewer parameters to choose from) and does not require extra metadata, and it might be the only choice if the other metadata is not available. Using unsupervised ML, one can learn about inherent patterns in the data, but it can be difficult associating these patterns with a particular phenotype, and metadata is obviously required in order to determine the actual capacity of the algorithms to assign samples into phenotypes of interest to the investigator.

In contrast, supervised ML (Table 2) requires examples of input–output data known as training datasets, i.e. genetic sequence to phenotype to learn from. In these types of algorithms, internal parameters are adjusted until the outputs of the algorithm resulting from processing the input training data match the true outcome values of the data as best as possible. For supervised ML, it is possible to accommodate outcome values that are binary (in a class or not), categorical (e.g. a host type) or continuous, depending on the algorithm. For this work, we consider either binary or categorical output values (classes) of host species, so here the task is one of training a supervised ML classifier.

After the training phase of supervised ML classification algorithms, new not seen before data can be processed and each entry is assigned an output value, or a probability of a certain output (class or label). During the training, an algorithm will learn specific patterns associated with each class. To assist the process of fitting the algorithm parameters to training data, some smaller part of the training dataset (usually 1/5 or 1/10) will be reserved while training is carried out on the rest of the data. This process (called cross-validation) will be iteratively repeated, accuracy noted on the output values of the reserved part and parameters adjusted until the best model (best internal parameters+best features describing each class) is decided.

All supervised ML uses the extra piece of information (the outcome values) in addition to the feature variables. For the ML classification tasks, we are considering this as a label, and the ML algorithms are designed to find commonality between data points with the same label even though these commonalities might not be the obvious ones. Here, we consider three different ML algorithms, which are suitable for the type of genotype–phenotype problems we are studying – these are SVMs (e.g. [13, 19]) neural networks (NNs) (see the reference by Drăghici and Potter [25] for an early example), and random forests (RFs) (e.g. [12, 26, 27]). SVMs look for non-linear combinations of the input features, such that dividing lines in an abstract multi-dimensional space between the different classes can be established. NNs also combine features by weighting them and then applying thresholds (or a function that gives low and high values) to the combined result. NNs typically have one or more layers (known as hidden layers) that do the combining and a final layer that transforms the hidden layer values into the output classification values. Deep learning (DL) models use NNs with multiple connected hidden layers [28]. RFs ([29], see also the paper by Qi [30] for a bioinformatics review) are somewhat different to the previous two methods; a RF is a collection of many decision trees, and each decision tree takes a set of feature variables and gives an output (if gene 1=A and gene 2=B then host=X; or if gene 1=D and gene 3=E then host=Y, etc.). But there are many trees and each tree could be using different features to reach a conclusion; hence, the final output of a RF is the summary of the individual outcomes (75 % of the trees reported the host was X, etc.).

Thus, with supervised ML new previously undiscovered patterns can be revealed, such as which set of genes or mutations are associated with a label, and this can be a very appealing part of certain models, especially RF, as they rank features based on relevance and these may signpost biology underlying the phenotype. However, with certain supervised approaches such as SVMs and NNs, it is harder to define which features are important, but many more features are included. As a consequence, these are sometimes referred to as ‘black box’ algorithms. It must be stressed that there is always the concern that not all discovered patterns are truly related to the particular phenotype; some might appear due to noise and/or biased sampling. To disentangle those features that are biologically relevant can be very challenging, even when they are true as they can be co-dependent; this is a general issue for understanding polygenic traits.

All of the above described techniques were used to address source attribution of *S. enterica* serovar Typhimurium. The results are focused on: (1) how each method performs according to known host information about the dataset; (2) how user-friendly the techniques are; (3) the interpretability of the results; and (4) any additional value of the technique.

## Methods

For this study, the dataset of 1203 *S *. *enterica* serovar Typhimurium genome sequences from our previous work [19] ⁠was reanalysed. The dataset is based on *S. enterica* serovar Typhimurium sequences from four hosts: 311 avian (A) isolates, 300 bovine (B), 336 human (H) and 256 swine (S). To explore the dataset and classify bacterial sequences into the four host categories, DRT as well as supervised and unsupervised ML methods were used (Table 2).

PVs associated with each host (*P*>0.05) were calculated by pangenome genome-wide association studies (GWAS) software Scoary [31]⁠ after 500 permutations. All calculations and visualization presented in this work were carried out with R studio. Significance tests were done using R implemented t.test{stats} and prop.test{stats}. Local polynomial regression fitting (loess{stats}) was used with 10 % smoothing span and fitted with generic predict{stats} function. Diversity was explored with R package ‘vegan’ from which the following functions and methods were used: ‘diversity’ for Shannon index calculations, betadiver(x, ‘z’), betadisper(), anova(), plot.betadisper(), permutest.betadisper(x, pairwise=T, permutations=99) for beta diversity analysis. Dissimilarity matrix for all unsupervised ML analysis was calculated based on Euclidean distance.

The script that shows packages, functions and parameters for each model used on this study is provided (Supplementary Material). Fivefold cross-validation (sliding window) was used for all supervised models, and the same splits of data were used in each algorithm. To assign host scores, one isolate at a time was removed from the training, the model has been trained and then the isolate tested.

SVM was run using R package e107. The initial data exploration indicated that the best performance can be achieved with radial kernel. To tune the model, we searched parameter space within following values gamma=10^(−6:−1), cost=10^(1 : 4) and a best performance for each model (each host) was determined. The classification in each model was done only for two classes a ‘host’ and ‘others’; therefore, after all isolates were predicted for one host, the labels were switched to the next ‘host’ and all remaining isolates became ‘others’. For the RF to test all isolates the same ‘leave-one-out’ training-test strategy was also used. The number of bootstraps and the depth of the trees were set to ntree=3000, mtry=100. During RF classification, two approaches were used: predict all four host labels at once, or iteratively predict only two classes at once (‘host’ and ‘others’). However, the results for these two approaches were identical.

## Results

### Diversity and Dimensionality Reduction

#### Measuring information content

Many different types of data (units for analysis) can be generated from sequences (Table 1). For example, different short sequence regions (k-mers) could be defined as a unit of difference, in our case we have opted for predicted proteins that are defined based on ORFs identified in the *de novo* assemblies. Proteins are an important 'operational unit' and therefore a logical unit for such differential analysis. As previously described [19], Roary [32] was used to generate a pangenome matrix that contains all the predicted proteins present in the isolates. In this case, it contained 23 307 clusters of PVs. The cut-off for defining a new variant was 95 % sequence similarity and 90 % of length. The mean number of predicted PVs for an isolate across the combined dataset was 4620 (min=4037, max=4993), while mean values of predicted PVs for each host were very similar with slightly less PVs in the human dataset (A=4632, B=4645, H=4573, S=4636). There was a significantly lower (*P*<0.001) number of core predicted proteins in the avian population (A=2218, B=3054, H=3056, S=3065) compared to the remainder. The total number of core protein clusters across all isolates was 1991, indicating only a partial overlap in proteins considered 'core' in each host. Rare PVs, defined as those found in less than 15 % of all isolates, originated the majority of the PV clusters (*n*=18 203) and this number of rare PVs actually varied very little by host (A=18 354, B=18 295, H=18 272, S=18 415). Overall, singletons generated 78 % of the whole pangenome, while the core was only 8.5 % of all PVs.

To examine the diversity of proteins between host species and, thus, get an idea of how much information proteins as features contain, we used the Shannon entropy measure [33]. Shannon entropy (also known as the Shannon index) is commonly used in information theory and signal processing [34], but is also extensively used as a biodiversity measure (for example, see the reference by Sherwin [35]). For a feature, here a PV, an index of 0 means no variation across the dataset, and the higher the index the more different alleles of the protein are present and the more ‘information’ that gene contains.

**Fig. 1.**
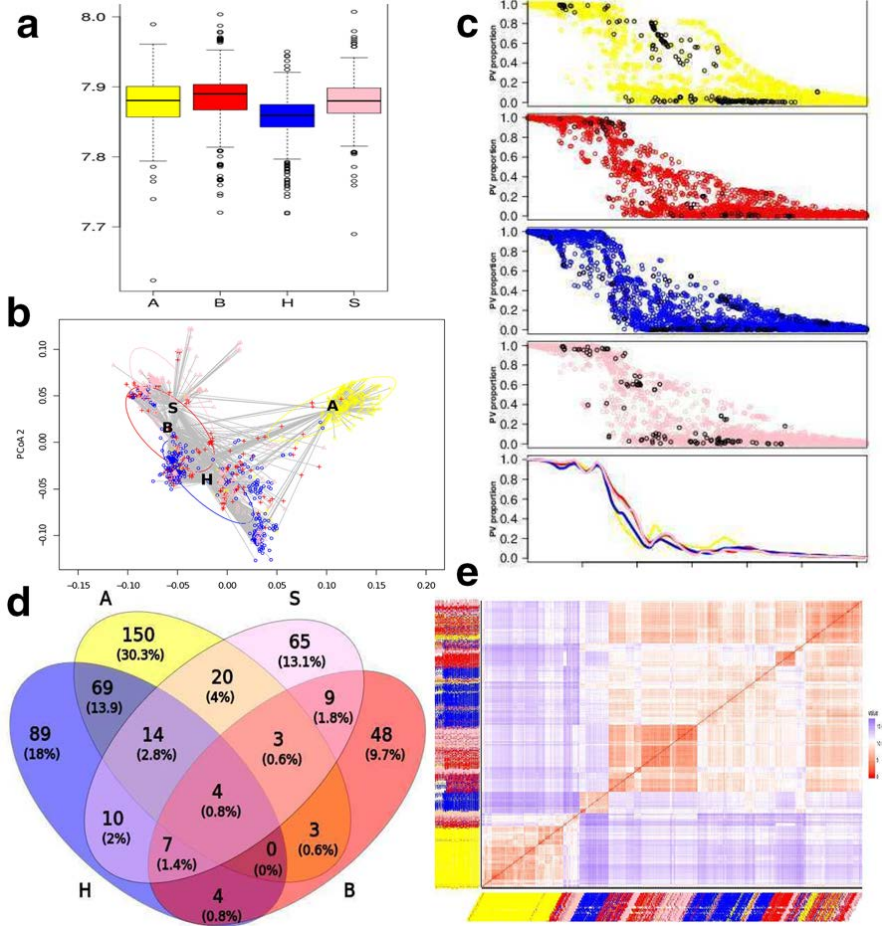
*
S. enterica* serovar Typhimurium pangenome exploration. Colours represent host: avian (yellow), bovine (red), human (blue), swine (pink). (a) Shannon index calculated for each host based on differential PVs. (b) Beta-dispersions show multivariate homogeneity of isolates in each host. Non-Euclidean distances between objects were reduced to principal coordinates (*x*-axis and *y*-axis). Ellipses indicate 1 sd from each host centroid marked as a letter. (c) 4041 PVs (*x*-axis) and the proportions (*y*-axis) of presence vary between the different hosts (colours as defined above). Those PVs that are significantly associated with each host (as calculated by pangenome GWAS, Scoary) are plotted in black. The bottom panel shows best fit lines (Loess) for distribution of differential PVs from all hosts. (d) Numbers of PVs significantly associated with host and overlap of differential PVs between the hosts. (e) Ordered dissimilarity matrix based on differential PVs. Heatmap colours: red (high) and blue (low) similarity. Labels are coloured by host.

Shannon indexes were calculated across all the predicted PVs and very similar levels of diversity were evident across the four isolation hosts; a mean value of 7.88, with only a slight decrease for the human dataset (Shannon index=7.85; Fig. 1﻿a). Another aspect of diversity can be described by beta diversity [36, 37]⁠ and it is used here to calculate a mean dissimilarity from individual observation units (PVs) to their host group. The clusters of PVs that resulted from this analysis, with mean distances to the centroid A=0.09725, B=0.10578, H=0.12157 and S=0.09975, were significantly different between hosts (ANOVA, *P*<0.0001) and in particular, between human and any other host (pairwise comparison with permutations, *P*<0.0001), see Fig. 1(c). Avian and swine isolates were gathered into the tightest PV clusters, while bovine and human sub-populations were more dispersed. Apart from the density, it can be noted that some PV clusters overlapped significantly, for example swine and bovine, while the majority of avian isolates were grouped distinctly. Based on this analysis, human isolates can be divided into two subgroups: one overlapping with the bovine group and another scattered between human and avian groups and mixed with isolates from other hosts. Another interesting observation is that data points were not gradually dispersed from the host groups. These isolates that are scattered away from the centroids usually appeared by more than 1 sd away (see Fig. 1b). Overall, while alpha diversity (Shannon index) was quite similar for each host, meaning the number of genes and their proportions were comparable, beta diversity indicates that the human isolates were different in their gene composition and proportions from all other hosts.

#### Feature selection

To identify genetic content that associated with each host, all clusters that were present in 100 % of isolates (i.e. core, *n*=1991) were removed, then the dataset was reduced further by removing all clusters that were present in equal proportions across different hosts, leaving only PV clusters for which the proportions between hosts varied. PVs that do not differ in proportion between their isolation hosts should have no predictive value in terms of host restriction. A PV is considered to be informative and, therefore, included even if its proportion differs in at least one host. The remaining differential 4041 PVs are shown in Fig. 1. As a whole, no significant differences in the distribution of PV proportions were identified in each host population as shown by best fit lines (Loess) (Fig. 1c). Proportions of the majority of the 4041 PVs only varied slightly between hosts (Fig. 1b); however, there were some PVs that were significantly associated with host groups as calculated by pangenome GWAS, Scoary [31]⁠. Furthermore, some of the PVs significantly associated with more than one host, i.e. a PV could be significantly over-represented in one host and at the same time could be significantly under-represented in another host (Fig. 1d). There were 263 avian significantly associated PVs and 113 (43 %) of these were shared between other hosts, 78 PVs were significantly associated with the bovine host from which 30 (38 %) were shared with other host groups. There were 197 significant human associated PVs with 108 (55 %) shared, and 132 significant swine PVs with 67 (51 %) shared. As some PV clusters were shared between hosts, the total number of unique significant differential PVs was 495. Each host's differential PVs have been plotted as black circles in Fig. 1(c) as well as in a Venn diagram to visualize the overlap of the PVs between each group (Fig. 1d). It is of note that the majority of the significantly differential PVs are due to lower proportions of the PV in a specific isolation host, while relatively few have higher levels in a specific isolation host: A=23, B=30, H=26, S=26.

All subsequent analyses in this study were applied to the reduced matrix of 495 PVs that represent only those flagged by the pangenome GWAS (Scoary) analysis as significant. Our process is, therefore, selecting the strongest differential features, as carried out within Scoary, which includes stepwise statistical analyses: (1) Fisher’s exact test; (2) Bonferroni and Benjamini–Hochberg adjustments; (3) corrections based on population structure; (4) random label permutation testing. Before any clustering analysis, it is useful to examine the clustering tendency of your data and this can be achieved by applying Hopkins statistics with a range between zero and one. Zero indicates uniformly distributed data and one absolute separation (highly clustered). The dissimilarity matrix based on Euclidean distances obtained from these 495 PVs showed some clustering (Hopkins statistics=0.25), which was very similar to that obtained by phylogeny, either based on SNPs or the accessory genome [19]⁠ (Fig. 1e). Therefore, despite strong selection by Scoary, the relationship between the 495 PVs and the fours hosts does not form a strong underlying structure.

Could dimensionality reduction by combining and transforming the selected features separate data into clear host-related clusters? If clear separation by host is achieved, this would indicate that the features could be reduced by combining them. Using principal component analysis (PCA), all 495 of the selected features contributed to a lesser or greater extent to the separation of isolates without any stand-out influencers. PCA does show that the majority of avian isolates can be separated by the first principal component (PC) from all other isolates (Fig. S1, available with the online version of this article). In addition, there is a small group of bovine and porcine isolates that are placed apart from the bulk of other isolates, as well as apart from the separated avian isolates, indicating that these are seen as a different sub-population separate from the remainder of bovine and porcine isolates (Fig. S1). However, there is considerable overlap for the majority of isolates from all other hosts that are placed as a large cluster of mixed isolates. As such, the complexity of the data can be seen by the percentage of variation explained by PCs, all three PCs account for only 46 % of variation (PC1=26 %, PC2=12 %, PC3=8 %). Therefore, we decided that an additional dimensionality reduction step prior to the application of the ML algorithms was unnecessary.

### Unsupervised ML

**Fig. 2.**
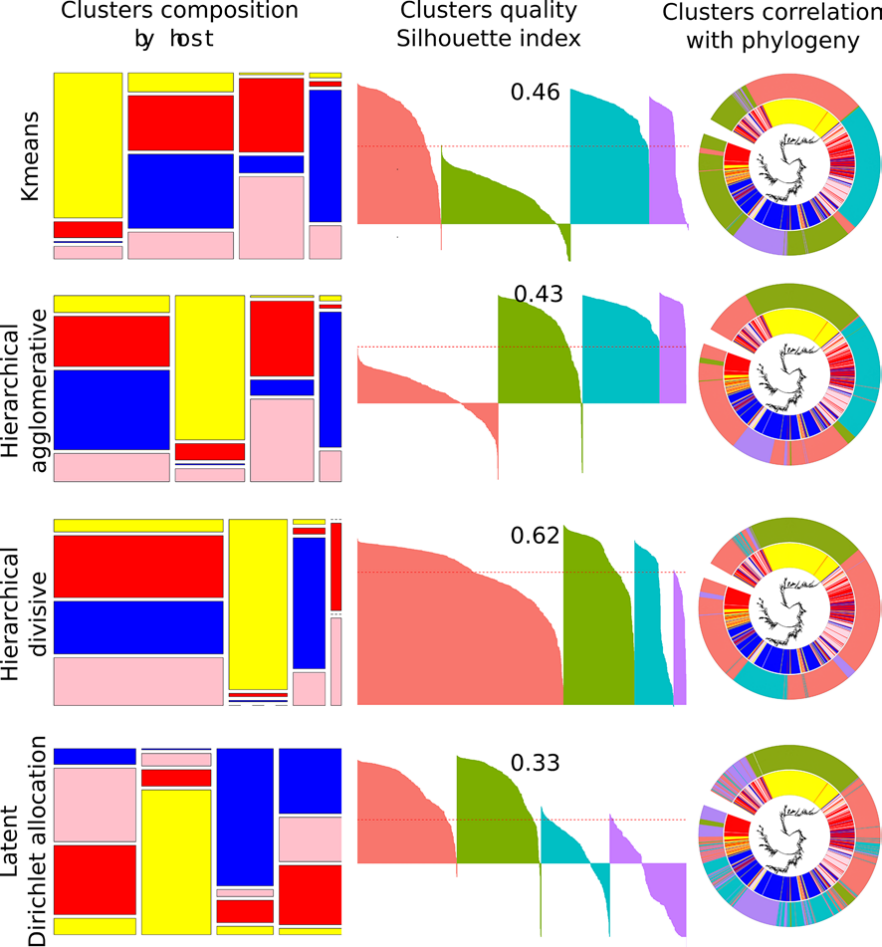
Unsupervised ML. The colours represent host: avian (yellow), bovine (red), human (blue), swine (pink). The first column of the figure shows the cluster’s relative size and composition by host. The second column demonstrates Silhouette index cluster assessment, where each of the four clusters are coloured differently and each isolate is drawn as a bar with its allocated value between −1 to 1. The mean value of all individual indexes is given on top of the silhouette cluster and is also denoted as a red dotted line through each graph. The clusters are drawn in the same order as those from the first column. The third column illustrates cluster correlation with phylogeny (accessory genome tree) with the inner ring depicting the host and the outer ring the unsupervised ML clusters based on the four group allocation.

Four methods of unsupervised learning were compared (Fig. 2): k-means, hierarchical agglomerative, hierarchical divisive and LDA (Table 2). Even though the results of DRT and unsupervised ML (clustering) seem to be similar, as they both split datasets into smaller subgroups, these two methods differ in that DRT aim to compress features, whereas clustering aims to group data points. Also, for clustering algorithms, it is often necessary to set the number of clusters that a user would like to obtain before running the analysis. Some techniques can indicate the optimal number of clusters for each of the unsupervised ML algorithms: this is done by: (1) computing different numbers of clusters and comparing within cluster 'sum of squares', sometimes called the elbow method; (2) the average silhouette method, which works out how well each point (isolate) lies within the cluster; or (3) gap statistics methods, which compare intra-cluster variation with their expected values under a null reference distribution [38, 39].

**Fig. 3.**
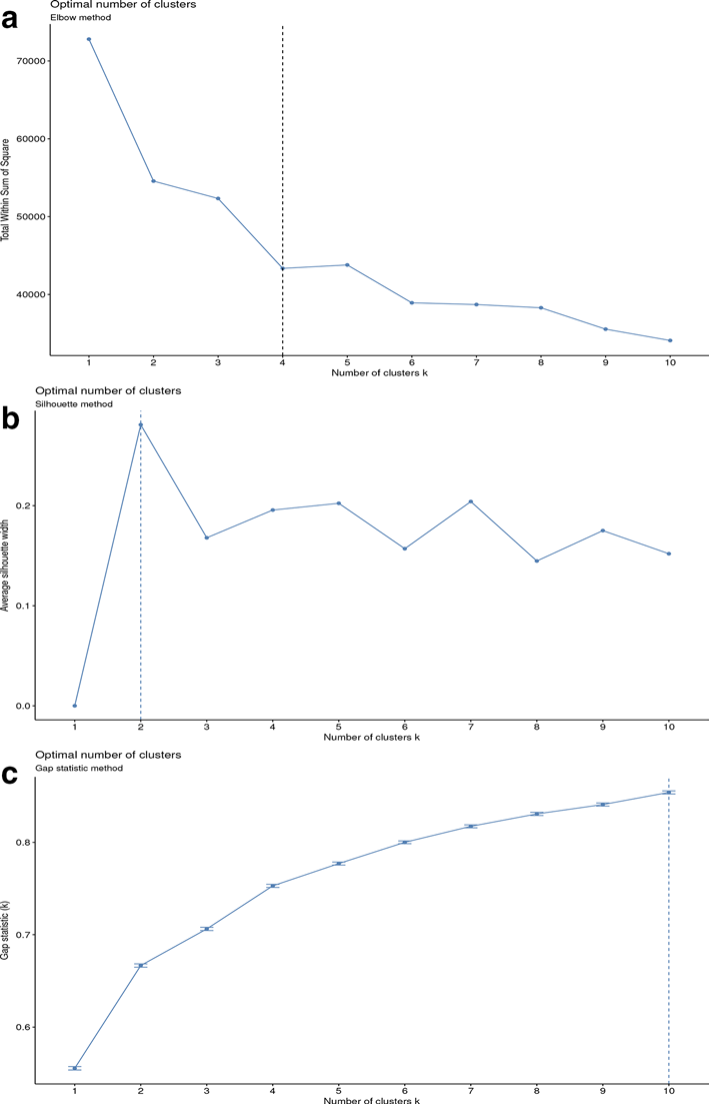
Optimal number of clusters as calculated by: (a) elbow method, (b) silhouette method, (c) gap statistic method. The methods that calculate the optimal number of clusters in the *S. enterica* serovar Typhimurium dataset were in disagreement and the recommended number of clusters ranged between 2 using the silhouette method to greater than 10 using GAP statistics.

All three cluster assessments were applied to demonstrate the number of clusters that would be considered optimal, based on k-means clustering. Fig. 3 demonstrates that each method comes to a different solution, and the number of optimal clusters varies from 2, as recommended by the silhouette method, to 10, when employing gap statistics. Moreover, according to the gap statistics graph, 10 is not yet the optimal number of clusters as the trend line has not yet reached a plateau. Overall, there was no agreement between cluster assessment methods, even though each of these produce stable solutions. All subsequent unsupervised ML analyses were based on only four clusters, as the objective was to assess how well this approach would assign the strains into the four original host species from which the bacteria were isolated.

Cluster assessment methods can be used not only in the beginning, to guide the analysis, but also at the end, to assess how well clustering algorithms have performed. There are over 30 different indices that could be used and recent studies [40]⁠ indicate that some of these, including silhouette, Davies–Bouldin and Calinski–Harabasz, perform the best in a wide range of situations. Thus, silhouette indices were used to measure the similarity between a data point inside of a cluster and those in other clusters. Silhouette assigns a score to each data point and these scores range from −1 to 1. The best clusters should have a mean score near 1. If the mean score is near 0, it could indicate that cluster members would be better separated into more, smaller clusters. When the value is negative, it is an indication that the data points were wrongly placed into this cluster.

The results of the clustering were also mapped onto the previously obtained maximum-likelihood phylogenetic tree [19]⁠, mainly because phylogeny is a well-established way to visualize bacterial sequence-based datasets and the diversity of the bacteria in question. It also allows inference about relationships between particular isolates with Bayesian methods applied to infer ancestry (see, for example, the work by Richardson and colleagues [41], which uses Bayesian phylogenetics [42] to examine host switching in *Staphylococcus aureus *). For the *S. enterica* serovar Typhimurium data and the relationship with host of isolation, it was evident that both the core and pan trees contain a 'clean' avian cluster ([19]; Fig. S2)⁠ that incorporated ~80 % of the avian isolates, while the other 20 % were spread across the tree and found in close proximity to isolates from other hosts. Based on the pangenome phylogeny, there is also a human cluster that contained ~50 % of isolates and a smaller bovine cluster with ~30 % of this group.

Overall unsupervised ML agreed in allocation of the majority of the isolates into particular clusters. (Fig. 2). So, all the unsupervised ML methods concluded that the majority of the avian isolates, also shown by phylogeny as related, should belong to the same cluster. Moreover, the human isolates were most of the time divided into three clusters, with one mainly human and two others of mixed host origin. It is intriguing that all unsupervised ML methods agreed that some of the phylogenetically close bovine isolates (on the right side of the phylogenetic tree in Fig. 2) were separated from the main bulk of bovine isolates and allocated to the avian cluster. Comparing four different unsupervised ML methods, it is evident that k-means and agglomerative hierarchical clustering finished with very similar solutions, with only 4.4 % of the sequences allocated to different clusters. A large mixed population cluster characterized all unsupervised ML results, with the majority of these isolates being the same, and coming from the left part of phylogenetic tree (see Fig. 2, column three, cluster correlation with phylogeny).

Silhouette indexes for unsupervised ML varied from 0.33 for LDA to 0.62 for HD; thus, based on that measure, HD had the most successful clustering strategy. k-means and HA both achieved very similar results and indexes of 0.46 and 0.43, respectively. However, the number of erroneously allocated isolates (silhouette index below 0) was higher with HA clustering. HD created the 'cleanest' avian cluster compared to all other unsupervised ML (with only two human and five bovine included in that cluster); however, HD generated the largest mixed population cluster that was composed of 754 isolates (67 % of all isolates).

The LDA method produced the most variable clusters. Apart from the 'mostly' avian cluster, its choice of the isolates for a particular cluster, when compared with their phylogeny, seemed much more segmented, indicating that this algorithm identified different and more granular patterns than the other algorithms. This is not especially surprising, since the LDA method is designed to find sets of patterns and isolates are not uniquely classified to a single pattern.

### Supervised ML

**Fig. 4.**
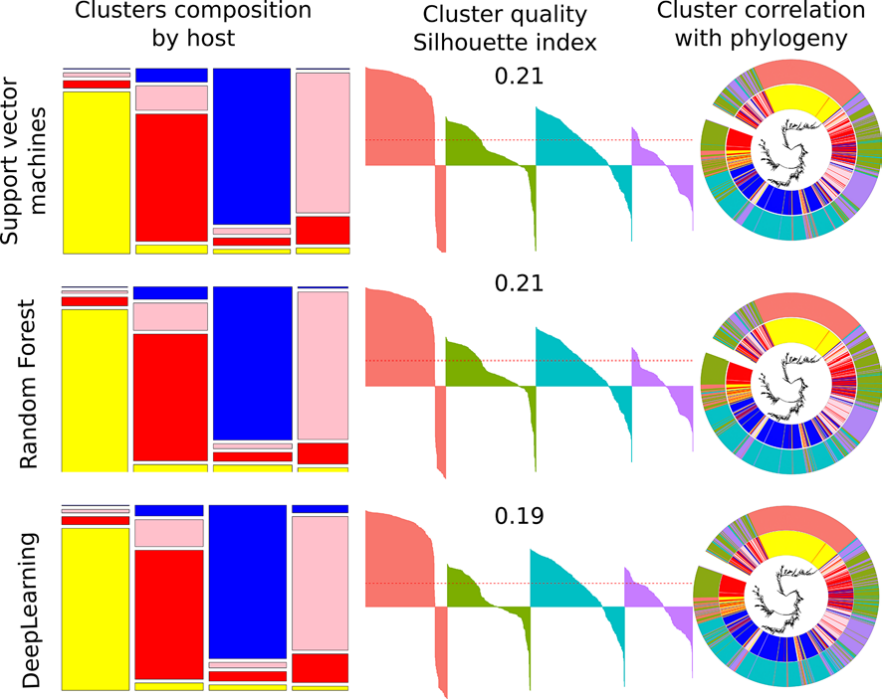
Supervised ML. The colours represent host: avian (yellow), bovine (red), human (blue), swine (pink). The first column of the figure shows the cluster’s relative size and composition by host. The second column demonstrates silhouette index cluster assessment, where each of the four clusters are coloured differently and each isolate is drawn as a bar with its allocated value between −1 to 1. The mean value of all individual indexes is given on top of the silhouette cluster and also denoted as a red dotted line through each graph. The clusters are drawn in the same order as those from the first column. The third column illustrates cluster correlation with phylogeny (accessory genome tree) with the inner ring depicting the host of isolation and the outer ring the supervised ML clusters based on the four group allocation.

The performance of SVM classifiers was demonstrated in our published research [19]⁠; however, the algorithm was reapplied to verify that the results were stable and could be repeated starting from the genome sequences, which was the case. In fact, all supervised ML methods examined here achieved very similar results, with ~80 % accuracy in the prediction of the bacterial source of isolation (Fig. 4).

Some variation during the training process was noted. SVM and RF cross-validation model accuracy were never higher than 80–85 %, while for the DL model the cross-validation accuracy could reach 100 %. Nevertheless, when isolates were tested with a 'leave one out' method, all algorithms showed very similar results with 85 % overall accuracy, with averaged accuracy by host: A=90.3%, B=78%, H=92% and S=75 %. The performance of all algorithms indicated that avian and human hosts contained the easiest to learn patterns, while bovine and swine hosts had many features in common and, therefore, were difficult to distinguish. Fig. 4 shows the performance of each supervised ML method with overall cluster composition and indication of assignment for each isolate. The tendency for errors is as follows: all hosts except avian have a second preferred group in terms of probable assignments. For human isolates this is the bovine group, for bovine it is swine and vice-versa. For avian, the erroneous assignments were spread equally between all other host categories (Table 3).

**Table 3.** Comparison of supervised ML methods by strain numbers assigned to each hostRow names A, B, H and S correspond to the actual host of isolation: avian, bovine, human and swine, respectively. Column names Ap, Bp, Hp and Sp, correspond to the predictions for these hosts.

**Method/host**	**Prediction**				
**SVM**	**Ap**	**Bp**	**Hp**	**S** **p**	**T** **otal**
A	278	16	9	8	311
B	13	234	15	38	300
H	1	25	309	1	336
S	7	45	12	192	256
Total	299	320	345	239	1203
**RF**				
A	275	15	15	6	311
B	15	240	18	27	300
H	1	24	309	2	336
S	4	52	10	190	256
Total	295	331	352	225	1203
**NN**				
A	281	13	11	6	311
B	14	226	19	41	300
H	1	19	305	11	336
S	6	47	11	192	256
Total	302	305	346	250	1203

High human scores for isolates from the non-human host group could indicate higher zoonotic potential for these particular isolates. Such scores are occasionally assigned by one or another supervised ML algorithm, but here we report only those isolates in which all three supervised ML algorithms agreed in the assignment to a human host. Eight avian isolates were called as 'human' by all three supervised ML methods, nine bovine and six swine.

According to silhouette index, the quality of clusters that were formed based on the prediction are of much worse quality than those from unsupervised ML, with a silhouette index 0.21 for SVM and RF and 0.19 for DL, reflecting that the silhouette index cannot capture the similarity of the patterns learned by supervised ML. Moreover, isolates that were allocated an index below 0 (*n*=365 for any of supervised ML methods), all except four were from the same host as the majority of isolates in that cluster.

## Discussion

The current availability of bacterial WGSs in public databases, as well as advances in computing capacity, enable retrospective quantitative population studies of diverse bacterial populations. The mosaic structure of particular bacterial genomes requires large datasets to be analysed in such comparative studies, and in turn this leads to increasing complexity of the datasets, which can be a challenge to interpret and visualize. Nevertheless, starting from bacterial WGS data, there is growing interest in the application of quantitative methods to predict phenotype from genotype. Phenotypes of particular interest are antimicrobial resistance and pathogenic potential to inform treatments and predict clinical outcomes. Another valuable outcome would be the capacity to predict the likely host of origin of an isolate, known as source attribution, which can be important in understanding the origins of an outbreak or contamination of a water source or food product. We have recently used an ML algorithm, SVM, to examine the source attribution of *Salmonella enterica* serovars, in particular *S *. *enterica* serovar Typhimurium. This serovar can be isolated from many different hosts, but there is now evidence that sub-clusters of *S. enterica* serovar Typhimurium may be host restricted [17, 18]. Accurate host assignment for something like *S. enterica* serovar Typhimurium is, therefore, still aspirational, as while we know the host of isolation for the sequences being studied, we do not know the extent to which the serovar is composed of generalist versus specialist strains.

This review aims to extend our previous work [19]⁠ by comparing additional statistical and ML methods for host source attribution, all analyses being based on the same dataset. For this comparative analysis, we started with a classical phylogenetic analysis of both a core SNP tree and a relationship tree based on the presence and absence of predicted PVs, which are based on ORFs from *de novo* assemblies. These two trees [19] showed quite similar results, with some clustering by host (Fig. S2 shows the relationships based on core SNPs); however, with obvious exceptions, many of the isolates still clustered to mixed host groups. Certainly phylogeny, while being an indispensable step in most bacterial genomics analyses, does not provide the resolution needed for host attribution with this dataset. However, it is remarkable that core and pan trees, while using very different information units (SNPs vs PVs presence/absence matrix), produced topologically quite similar trees [19]. These similarities between core and accessory could indicate that some loss, acquisition and maintenance of specific accessory genes may require a specific type of core background leading to such relationships. While this study has generally explored only information based on PVs, we consider that combining analyses with multiple information units (SNPs, k-mers, PVs) is likely to add power to decipher phenotype from genotype relationships.

We next explored some methods that can be applied to genomics data to measure diversity in terms of richness and evenness of the PV distributions from each host (alpha diversity) and between hosts (beta diversity). Differences between hosts were based on small changes in the proportions of PVs present; however, their cumulative effect leads to the conclusion for this dataset that the avian sup-population is the most diverse, while the PVs derived from the human isolate set were the most divergent from all other isolates. As with many conclusions from this study, which is presented mainly to consider the different methods, further comparisons between different datasets is needed to find out whether these observations are consistent and not a product of the analysis being on a biased sub-population.

Phenotype from genotype studies, i.e. GWAS, generally use large cohorts of unrelated individuals and assess associations between DNA variants and particular traits, such as disease phenotypes. The most commonly used variants are SNPs, although other types, for example, copy number variants, can also be used. Many bacterial species have become dependent on horizontal gene transfer to accelerate evolution and increase the chances of species survival. Consequently, pangenome GWAS may be more indicative of host adaptation than SNP-based GWAS. However, in this analysis there were relatively few PVs significantly associated with each host, ranging from 263 PVs in the avian isolates to 78 PVs from the bovine isolates, as determined statistically using Scoary [31]. Based on this step and additional analyses, the avian group possessed a significantly larger number of unique features, and as a consequence we see distinctive clustering of the majority of avian isolates by all of the algorithms used in this study.

Even the dramatically reduced PV sets produced by GWAS still represent a daunting task if required to describe and predict a particular host population. Usually advised as an early exploratory analysis step, PCA was used to assess the co-linearity of these variants, as well as to identify more prominent features. The dataset is complex as the first three PCs explain less than 50 % of data variance. Therefore, for this particular dataset, no meaningful insights could be taken from this analysis as none of the existing features play a predominant role and the majority of the features contribute equally for the main PCs.

This was then followed by a consideration of some unsupervised ML methods; for this, ideally, the number of clusters should be decided beforehand. All of the three techniques that were used to decide the number of clusters produced different results, suggesting again that the data is complex and no obvious clustering solution could be identified. However, when all the unsupervised ML methods were asked to divide the data into four clusters (for the four hosts), all the approaches came to a very similar solution, confirming that there is a stable underlying structure in the dataset; however, this was not completely related to the host. To some degree, the association achieved by all unsupervised ML methods aligned with the phylogeny, which is in essence divisive hierarchical clustering with some modifications. We appreciate that even the same host population could be heterogeneous, so by dividing the dataset by more than four clusters we hoped to see multiple 'cleaner' clusters of the same host.

We experimented with dividing the dataset into between 2 to 30 clusters and even though, with increasing numbers of clusters, multiple clusters could be composed almost entirely of isolates from a single host, the solutions didn't seem to be stable, each time producing compositionally different clusters (data not shown). Clustering with smaller numbers of clusters generated more stable solutions with less than 2 % of isolates changing clusters between runs. In common with the previous methods, unsupervised ML clustered the avian group distinctly and otherwise had mixed host population clusters. This could indicate that these are true generalist strains, and not really different by gene content and, therefore, have equivalent capacities to survive in multiple hosts.

Three different supervised ML were tested and all arrived at remarkably similar solutions, with the majority of the isolates assigned correctly to their host of isolation and those that were assigned ‘erroneously’ to different hosts were mostly the same between the different supervised ML methods. Therefore, in terms of accuracy, for this particular task, bacterial host attribution for a medium size dataset, there were no major differences between the supervised ML algorithms. Nevertheless, there are always specific considerations when choosing a supervised ML algorithm for a task. SVM has the following advantages: high accuracy, strong theoretical guarantees regarding outfitting and with an appropriate kernel it can work well even if the data isn't linearly separable in the base feature space. However, SVM is usually memory intensive, needs specialist knowledge to run and tune, and very often predictions are based on very many features (support vectors).

By comparison, RF is the most user-friendly algorithm that has already been implemented in multiple R packages, as well as in Python and other scientific programming languages. There are three major advantages to RF compared to SVM: (1) it can predict multiple classes simultaneously; (2) there is no need to filter features because the bootstrapping capability will return a list of the most relevant ones, usually the list of features on which RF-based predictions is based is much shorter than that for SVM; (3) there are only a few straightforward parameters to adjust.

NNs and consequently DL, which is based on NNs with a substantial number of layers, are very promising approaches that have started to dominate the 'big data' field. There are many NN flavours each suited to different tasks, i.e. recurrent NN for word prediction and convolutional NN for image processing. The algorithms are highly scalable, which is a key quality nowadays when each new study results in an ever-increasing dataset. The time and computational restrictions become very evident even with such a relatively small dataset as analysed in this study, so if planning to analyse thousands of bacterial isolates then DL could be the right choice. Deploying NNs requires specialist knowledge and careful choice of multiple parameters. Even its shape, how deep and how wide your NN is, will influence the results. However, when implemented well with a good understanding of the underlying data, then the established NN can be reused with addition of new top layers while the underlying structure is kept intact. This strategy allows both time and computational savings as well as retention of previously learned patterns.

Over-fitting, the situation when an algorithm learns patterns specific to the dataset, but not relevant to the question in general, is the primary concern to any supervised ML study. One of the ways to increase confidence that the method is using features of biological relevance is to increase and diversify the dataset in order to blend out any confounding signals, i.e. where possible the training dataset should be gathered from different locations and times and in similar numbers. In the case of bacterial sub-population analyses, phylogeny should be included to ensure that the isolates come from different phylogenetic branches. A counter-argument against using a large mixed dataset is that different populations geographically may have evolved different gene groupings to support growth and survival in similar niches, including hosts. As such, by combining these sub-populations worldwide trends can be discovered but local adaptation missed; this is especially relevant for bacteria that are under relatively rapid genomic change, and potentially both global and local datasets should be studied in parallel. We acknowledge that in this study and in previous work [19], feature selection and training were carried out on the same population of isolates. As such, better testing of the model would require the application of these selected features on a new similar population. Furthermore, to test the biological value of significant PVs, there needs to be research in the respective host species comparing the colonization of isolates that have very different prediction scores.

Subsequent to our initial publications [13, 19], there have been two significant studies making use of ML methods for *Salmonella enterica* host attribution [12, 26]. In the Wheeler *et al*. study [12], a novel hidden Markov model-based approach (DeltaBS) was used to predict functional variation in protein-encoding genes among *S. enterica* lineages. The method assumes that variation in more conserved positions are more likely to impact protein function. DeltaBS was applied to the genomes of strains known to be associated with invasive or intestinal salmonellosis and supervised ML (RF) applied to accurately predict invasive potential. It was also able to differentiate recently emerged *S. enterica* serovar Typhimurium and *S *. *enterica* serovar Enteritidis lineages associated with invasive non-typhoidal *Salmonella* in sub-Saharan Africa [12]. The second study by Zhang *et al*. [26] focused on *S. enterica* serovar Typhimurium and applied a RF classifier for source prediction and this was able to correctly assign the host source for major zoonotic outbreaks characterized in the USA in the last decade. Their dataset deliberately selected for diversity across subgroups, while our analysis worked with the commonalities of the human infection situation (whilst excluding closely related isolates from outbreaks). We consider that as a consequence of this, we have a higher proportion of isolates from humans that do not have a high score for an alternative host. This difference may be due to the fact that the more common groupings of human *S. enterica* serovar Typhimurium infections may not be originating from other hosts, but clearly this requires further research. Taken together though, all of these studies [12, 13, 19, 26] affirm the value such supervised ML approaches can bring to attributing bacterial source and threat to human health.

In summary, and as anticipated, supervised ML was effective at being able to accurately attribute the host of isolation of *S. enterica* serovar Typhimurium based on WGSs. This analysis clearly shows that the *S. enterica* serovar Typhimurium population even within one host is not homogeneous. The multiple methods indicated that there are both specialist *S. enterica* serovar Typhimurium strains that group to a specific host, as well as isolates that ended up in mixed clusters with high scores for multiple hosts (generalists). The biological relevance of the attribution scores still needs to be studied, but the fact that multiple and diverse approaches demonstrated a large and distinct avian *S. enterica* serovar Typhimurium population (including phylogeny) means the existence of other such 'specialists' in the other hosts is more likely and has been shown for some human-restricted *S. enterica* serovar Typhimurium sub-types. ML encompasses powerful methods that allow analysis of complex datasets and these will have an increasingly important role to play in predicting phenotypes influenced by large numbers of genes, the pool of which can also vary depending on the epidemiology and genetic exchange mechanisms of the bacteria being studied.

## Data bibliography

1. Lupolova N, Dallman TJ, Holden NJ, Gally DL. Data used for this work can be downloaded from https://figshare.com/s/7a3ededa8cedd95b9fb7. The files include isolate IDs, PVs and their annotations for *Salmonella enterica* and *Escherichia coli* (2017).

2. Lupolova N, Dallman TJ, Holden NJ, Gally DL. Descriptive PVs for each original SVM model also can be found at https://figshare.com/s/7a3ededa8cedd95b9fb7. The name of the file describes the model for which these PVs were used. So, salmonella_PV_30_AO_annotations.csv means these are the PVs that describe *S. enterica* serovar Typhimurium avian isolates versus all other isolates (2017).

3. Lupolova N. Isolate metadata for both species (original host, predictions, place, year and multilocus sequence type) are visualized using pangenome trees and can be viewed on iTOL: http://itol.embl.de/shared/nlupolova (2017).

## Supplementary Data

Supplementary File 1
