# Supplementary material for: A guide to machine learning for bacterial host attribution using genome sequence data
Source: Microb Genom. 2019 Nov 28;5(12):e000317. doi: 10.1099/mgen.0.000317 (PMC6939162; doi:10.1099/mgen.0.000317)
Supplement: Supplementary File 1 [file mgen-5-317-s001.pdf]

Supplementary file

A guide to machine learning for bacterial host attribution using genome sequence data

N. Lupolova, S. Lycett, David L. Gally

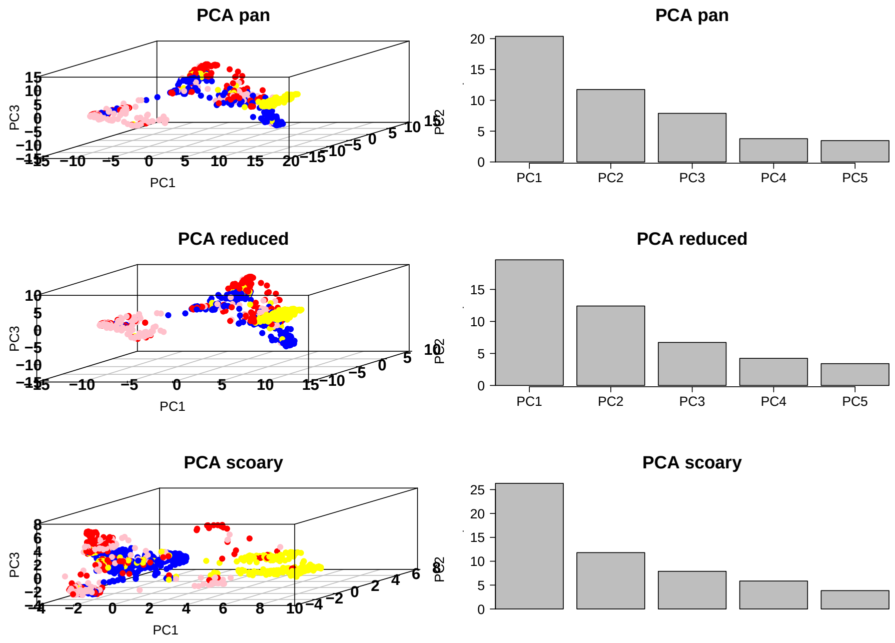

Figure S1: **Principal component analysis.**Principal component analysis (PCA) applied to pan genome protein clusters (PVs) of 1203 bacterial isolates. Colours represent isolate's host: avian (yellow), bovine (red), human (blue), swine (pink). PCA has been applied on the whole dataset of 23,307 PV clusters (top panel), dataset that contain only differential PVs (4,041 PVs) (middle panel) and data set with statistically significant PVs (495) as calculated by Scoary (bottom panel).

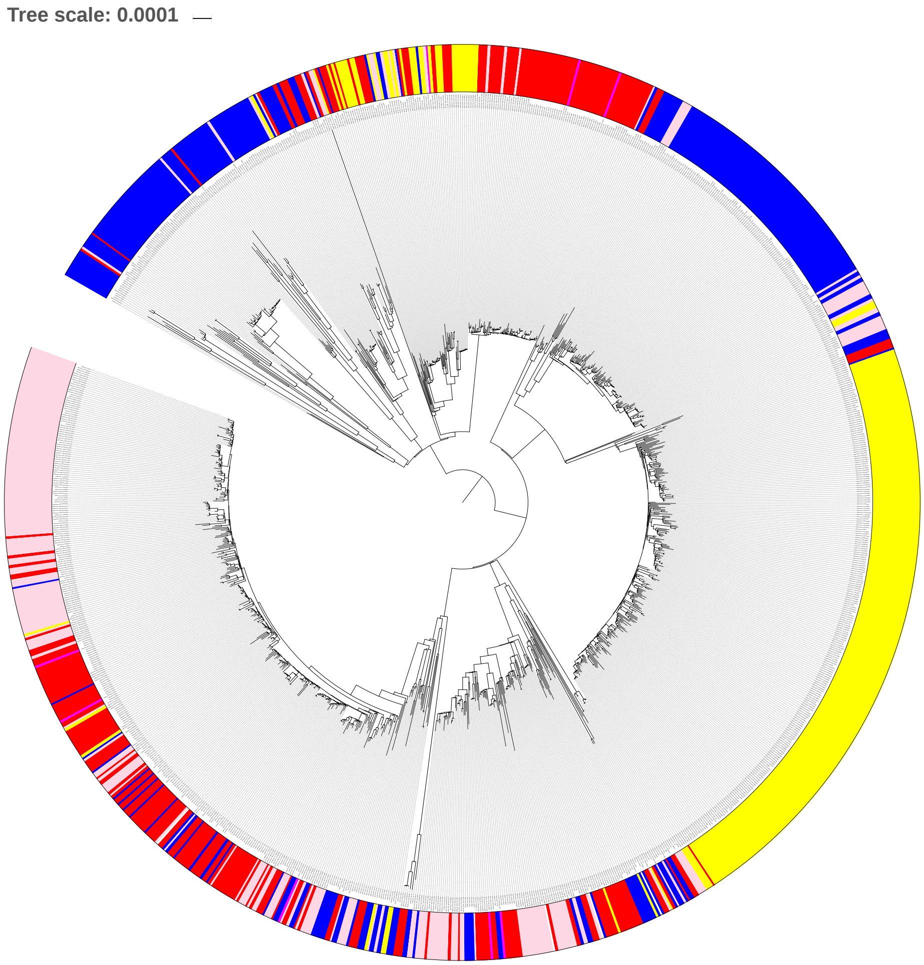

Figure S2: **Core genome phylogeny.** Core SNPs tree for S. Typhimurium isolates analysed in the study. The colours represent the host of isolation: avian (yellow), bovine (red), human (blue), swine (pink). Clear sub-clusters relating to host are present but there are also sub-clusters that contain isolates of variable origin.

Supplementary data script

```
# This script is a supplementary material and provides final list of
libraries, functions and parameters used for supervised and
unsupervised learning in the original research paper "A guide to
machine learning for bacterial host attribution using genome
sequence data".
library("factoextra")
library("cluster")
library("gdata")
library("e1071")
library("data.table")
library("LCA")
library("vegan")
library("dendextend")
library("h2o")
library("randomForest")





# load data
stm_scoary---------------------------------------------------------
----
stm_scoary<- read.csv("stm_scoary.csv", header = TRUE, sep=",")
stm_scoary <- data.frame(stm_scoary[,-1],
row.names=make.names(stm_scoary[,1], unique =TRUE))

# add extra column with colours for easy plotting afterwards and as
your labels for sML



A<-c(which(startsWith(rownames(stm_scoary), "typhimurium_a")))
B<-c(which(startsWith(rownames(stm_scoary), "typhimurium_b")))
H<-c(which(startsWith(rownames(stm_scoary), "typhimurium_h")))
S<-c(which(startsWith(rownames(stm_scoary), "typhimurium_s")))


stm_scoary["host"]<-NA
stm_scoary$host[A]<-"yellow"
stm_scoary$host[B]<-"red"
stm_scoary$host[H]<-"blue"
stm_scoary$host[S]<-"pink"
stm_scoary$host<-as.factor(stm_scoary$host)




# kmeans  ---------------------------------------------------------

set.seed(21)
```

```
kmeans_scoary<- kmeans(stm_scoary[, -length(stm_scoary)], centers=4,
iter.max = 100, nstart = 20,  algorithm = "Hartigan-Wong", trace=T)


## assess kmeans by silhouette
-----------------------------------------------

dissE <-daisy(stm_scoary[, -length(stm_scoary)])
dE2   <- dissE^2
sk1   <- silhouette(kmeans_scoary$cluster, dE2, Ordered =TRUE)
#


#aglomerative clustering----------------------

dendA <- as.dendrogram(agnes(dissE, method="ward"))

#divisive clustering------------------------
dendD <- as.dendrogram(diana(dissE, stand=F))



# topics_lda
--------------------------------------------------------------

library(topicmodels)


#Set parameters for Gibbs sampling
burnin <- 4000
iter <- 2000
thin <- 500
seed <-list(1,450,800,1001)
nstart <- 4
best <- TRUE
k<-4

ldaOut <-LDA(stm_scoary[, -length(stm_scoary)] ,k, method="Gibbs",
control=list(nstart=nstart, seed = seed, best=best, burnin = burnin,
iter = iter, thin=thin))

# svm --------------------------------------

#Here is "one against others" approach,  therefore change "test" and
"others" for each host acordingly. It is slow, in part  because of
the tunning.  Better submit jobs in parallel and for each host
independently. Also, to speed up the process, tune and search
parameters can be done only once in the beginning. However, not
tunning the model would decay performance.
test=c(A)
others=c( B, H, S)
probs<-c()

stm_scoary["host"] <- NA
```

```
stm_scoary$host[test]="A"
stm_scoary$host[others]="O"
stm_scoary$host <-  as.factor(stm_scoary$host)




for (i in range(1:nrow(stm_scoary))){

  testidx <- i

  svm_train = stm_scoary[-testidx , ]
  svm_test = stm_scoary[testidx , -length(stm_scoary) ]

#assign weight to classes as now they are  unbalanced

  wts<-100 / table(svm_train$host)

  #tune <- tune.svm(host~., data=svm_train, gamma=10^(-6:-1),
cost=10^(1:4), class.weights = wts)
  #summary(tune)
  # Majority of the parameter search attemps resulted in the best
gamma= 1e-04 and best cost=10
  set.seed(21)

  model = svm(host~., data=svm_train, cross=10, method="C-
classification", kernel="radial", probability=T,gamma= 0.001,
cost=10, class.weights = wts)


  svm_prediction = predict(model, svm_test[, -length(svm_test) ],
probability=T)

#check performance, use similarly for all other supervised ML
  #conf_matrix<- table(svm_test$host,svm_prediction )
  #print(conf_matrix)
  #print (probs)
  #print(model$tot.accuracy)


#gather predictions
  probs<-c(probs, round(attr(svm_prediction,"probabilities" ), 3))

}

#randome forest---------------------------------
  #reasign all 4 host, because in RF and NN more than 2  classes
can be used at once
  stm_scoary["host"]<-NA
  stm_scoary$host[A]<-"yellow"
  stm_scoary$host[B]<-"red"
  stm_scoary$host[H]<-"blue"
  stm_scoary$host[S]<-"pink"
  stm_scoary$host<-as.factor(stm_scoary$host)
```

```
probs<-c()
for (i in range(1:nrow(stm_scoary))){

    testidx <- i

   rf_train = stm_scoary[-testidx , ]
   rf_test = stm_scoary[testidx , -length(stm_scoary) ]

#do some testing and tuning

#test_var<-rfcv(trainx=rf_train[, -length(colnames(rf_train)) ],
trainy=rf_train$host) )
#tune<- tuneRF(x=rf_train, y=rf_train$host,  stepFactor=3, improve=
TRUE,  trace=TRUE, plot=TRUE, doBest=TRUE)

model <- randomForest( x=rf_train[, -length(colnames(rf_train)) ],
xtest=rf_train[, -length(colnames(rf_train)) ], y=rf_train$host,
ytest=rf_train$host , importance=TRUE, ntree=5000,
keep.forest=TRUE, mtry= 50)

probs <-c(probs, predict(model, rf_test, type="prob"))
}


  #neural network-------------------------



  h2o.init(nthreads = -1) #use all avalible cores
  stm_for_h2o<-as.h2o(stm_scoary)

  probs<-c()
  for (i in range(1:nrow(stm_for_h2o))){

    testidx <- i

    h2o_train = stm_for_h2o[-testidx , ]
    h2o_test = stm_for_h2o[testidx , -length(stm_for_h2o) ]

    # use of the simplest model which for this dataset gives the
best results
    stm_for_h2o.dl <- h2o.deeplearning(x = 1:(ncol(h2o_train)-1), y
= ncol(h2o_train), training_frame = h2o_train, shuffle_training_data
= T, seed =21)

    probs <-c(probs,  h2o.predict(stm_for_h2o.dl, h2o_test))
  }

  h2o.shutdown() # disconect
```
